# Differential Signals From TNFα-Treated and Untreated Embryos in Uterine Tissues and Splenic CD4^+^ T Lymphocytes During Preimplantation Pregnancy in Mice

**DOI:** 10.3389/fvets.2021.641553

**Published:** 2021-03-08

**Authors:** Katarzyna Buska-Mach, Anna Ewa Kedzierska, Adam Lepczynski, Agnieszka Herosimczyk, Małgorzata Ozgo, Pawel Karpinski, Agnieszka Gomulkiewicz, Daria Lorek, Anna Slawek, Piotr Dziegiel, Anna Chelmonska-Soyta

**Affiliations:** ^1^Hirszfeld Institute of Immunology and Experimental Therapy, Polish Academy of Sciences, Wrocław, Poland; ^2^Department of Physiology, Cytobiology and Proteomics, West Pomeranian University of Technology, Szczecin, Poland; ^3^Department of Genetics, Wroclaw Medical University, Wrocław, Poland; ^4^Department of Human Morphology and Embryology, Wroclaw Medical University, Wrocław, Poland; ^5^The Faculty of Veterinary Medicine, Wroclaw University of Environmental and Life Sciences, Wrocław, Poland

**Keywords:** embryo, TNF, uterus, pregnancy, CD4 lymphocytes, preimplantation, proteome, T cells

## Abstract

The main aim of this study was to examine if a female mouse body in preimplantation pregnancy can distinguish between embryos of normal and impaired biological quality in the local and peripheral compartments. Normal (control group) and TNFα (tumor necrosis factor-α)-treated embryos (experimental group) at the morula stage were non-surgically transferred into the uteri of CD-1 strain [Crl:CD1(Icr)] female murine recipients. Twenty-four hours after the embryo transfer, females were euthanised, and uteri and spleens were dissected. In uterine tissues (local compartment), we assessed the expression of 84 genes comprising nine signal transduction pathways, using a modified RT^2^ Profiler PCR Array. In the spleen (peripheral compartment), we determined the proteome of splenic CD4^+^ lymphocytes using 2D protein electrophoresis with subsequent protein identification by mass spectrometry. Sample clustering and differential gene expression analyses within individual signal transduction pathways revealed differential expression of genes in the uteri of females after transplantation of normal vs. TNFα-treated embryos. The most affected signal transduction cascade was the NFKB (Nuclear factor NF-kappa-B) pathway, where 87.5% of the examined genes were significantly differentially expressed. Proteomic analysis of splenic CD4^+^ T lymphocytes revealed significant differential expression of 8 out of 132 protein spots. Identified proteins were classified as proteins influenced by cell stress, proteins engaged in the regulation of cytoskeleton stabilization and cell motility, and proteins having immunomodulatory function. These results support the hypothesis that even before embryo implantation, the body of pregnant female mice can sense the biological quality of an embryo both at the local and peripheral level.

## Introduction

The maternal recognition of the embryo is essential during implantation for the establishment of pregnancy and its maintenance. During the preimplantation period of the pregnancy paracrine signaling is the main way of interaction between the mother and the embryo. At this time, the uterine mucosa is strongly influenced by steroid hormones, which change the secretory activity of the endometrium and ensures optimal development of the embryo (1–3). On the other hand, the presence of an embryo in the uterus itself modulates the metabolism of endometrial tissue by influencing the expression of genes involved primarily in tissue remodeling and immunological processes (3). The presence of the embryo in the uterus before implantation is also signaled outside the tissues of the reproductive system. This process is particularly marked in cells of the immune system (4, 5). Additionally, recent work by Behura et al. (6) showed that the expression of genes of the receptors and their ligands in uterine and brain tissues at the preimplantation period of pregnancy is related. The mechanism of signal transmission from the uterus to the brain remains unknown. On the other hand, the neural connections of the sympathetic and parasympathetic systems and the sensory fibers with immune cells at the periphery indicate that signal transmission between distant compartments of the body may be guided by neurochemical conduction (7). The functional significance of these links needs further investigation. Nevertheless, it can be assumed that the early embryo-uterine-immune and nervous system interaction underlie the fundamental mechanisms of pregnancy maintenance, including fetal immune tolerance (4, 8), and also still not the well-recognized mechanism of brain control of early pregnancy.

During the pre-implantation phase not only embryo recognition takes place. It is a moment of critical decisions that are being made by a pregnant female: continuation of gestation or its rejection. In humans, pregnancy rejection could be expressed by the incidence of early embryo death and pregnancy loss and is estimated to constitute ~30% of known pregnancies (9). A similar phenomenon is observed in pigs and cattle, where approximately 30% of early pregnancies are lost (10, 11). This high embryo death rate before implantation signifies the result of an intensive embryo selection process. This process is dependent on mutual interactions between the mother and embryo, and its dysfunction leads to the survival of unwanted embryos with low biological competence. In turn, this situation results in an increased abortion rate in more advanced stages of pregnancy (12). Therefore, proper assessment of the biological competence of an embryo by the pregnant female is critical for avoiding an unnecessary energy investment and maternal stress associated with pregnancy failure. The preimplantation embryos manifest their presence both in local and peripheral compartments of the immune system. Our previous work has shown that locally, in the uterus, the presence of the embryo before implantation selectively affects the murine gene expression regulated by signal transduction pathways. In the majority of signaling pathways in pregnant female mice compared to females in pseudopregnancy down-regulation of the uterine gene expression was observed (13). Our results are similar to those reported by other authors, who have shown that normal pregnancies modulate gene expression in the endometrium before the trophoblastic invasion (14–20). It is worth noting that gene expression of some signaling pathways in horses, pigs, and cattle, mainly those induced by interferons, is conservative, which indicates common mechanisms of pregnancy recognition in mammals (21). Existing studies have revealed that *in vitro* cultured human decidual cells can distinguish the biological quality of embryos (12). Therefore, it is suggested that human decidua may act as a natural biosensor of the developmental potential of pregnancy. Although an early foeto–maternal dialogue in the uterus is well-documented, the recognition and response of the maternal peripheral immune system to the presence of fetal antigens remains unknown. It is still debated whether maternal peripheral leukocytes' awareness of conceptus antigens is an important event for the establishment of a pregnancy-related immune alteration. Nevertheless, peripheral Th1/Th2 switch during pregnancy and diminished frequency of transgenic T lymphocytes specific for male H-Y antigens in pregnant females with male fetuses indicate pregnancy-induced immunomodulation (22). This observation points to the ability of lymphocytes to recognize pregnancy-related antigens at the periphery. Moreover, in BALB/c females mated with transgenic males that express a green fluorescent protein (GFP) the presence of GFP DNA was confirmed in the spleen, blood, and some maternal organs beginning on 0.5 days *post coitum* (dpc). GFP expression was under the control of the β-actin promoter (homozygous, C57BL/6 background). This observation is another evidence, which confirms the presence of paternal antigens in maternal tissues during early pregnancy (23) and suggests the possibility of their immune recognition. Besides, our previous work showed that splenic antigen-presenting cells (APCs) change their costimulatory phenotype in preimplantation pregnancy (24, 25). Also, we revealed that the presence of a preimplantation embryo changes the proteome of splenic T CD4^+^ lymphocytes in pregnant female mice compared to pseudopregnant mice (26). These examples support the hypothesis that the maternal peripheral immune system recognizes early pregnancy and is able to adjust its immunophenotype to a new semi-allogeneic antigen challenge. On the other hand, in the preimplantation period of abortion-prone pregnancy (CBA/J♀xDBA/2J♂), significant differences in the frequency of female splenic regulatory T lymphocytes are observed in comparison to normal pregnancies (27). This finding suggests that the process of development of peripheral immune tolerance is different during the early stages of normal and compromised pregnancy.

Here, we studied the expression of genes related to uterine pathways of signal transduction and protein expression in peripheral lymphocytes in female recipients of normal and TNFα-treated embryos. TNFα has adverse effects on early embryonic development. It induces stable phosphatidylserine redistribution in the plasma membrane of embryos. Phosphatidylserine redistribution is believed to be an early signal in the process of apoptosis. It also significantly increases the incidence of apoptotic cells in blastocyst (28). However, TNFα-treated embryos continued their development to the stage of the hatched blastocyst. What is important is that after the transfer into recipient mice, TNFα pre-treated blastocysts got implanted at approximately the same rate as control embryos did (28). Nevertheless, the rate of resorption among fetuses after exposure to this cytokine increased significantly (28, 29). Therefore, TNFα-treated embryos can be described as developmentally compromised, yet implantation-competent.

Taking into consideration all the aforementioned mechanisms of early recognition of preimplantation pregnancy, we wondered whether TNFα-treated embryos with a compromised developmental potential may signal their presence differently from normal embryos under *ex vivo* conditions both in the local and peripheral compartment. For this purpose, we screened the expression of uterine genes involved in signal transduction pathways (local compartment) and protein expression in splenic lymphocytes (peripheral compartment) in female recipients of normal or TNFα-treated embryos.

## Materials and Methods

### Animals

Outbred CD-1 [Crl:CD1(Icr)] mice purchased from Charles River Laboratories (Sulzfeld, Germany) were housed under specific pathogen-free (SPF) conditions on a dark–light cycle 12:12. All surgical procedures were performed under isoflurane anesthesia and meloxicam analgesia. Every effort was made to minimize the animals' suffering. All the animal experiments were approved by the Local Ethics Committee for Experiments on Animals at the Hirszfeld Institute of Immunology and Experimental Therapy in Wroclaw (approval No. 41/2010).

We studied two groups of femal mice (*n* = 10 in each group): after non-surgical transfer of normal embryos (NE) or TNFα-treated embryos (TNE).

### Female Embryo Donors and Collection of Embryos

**Figure 1:**
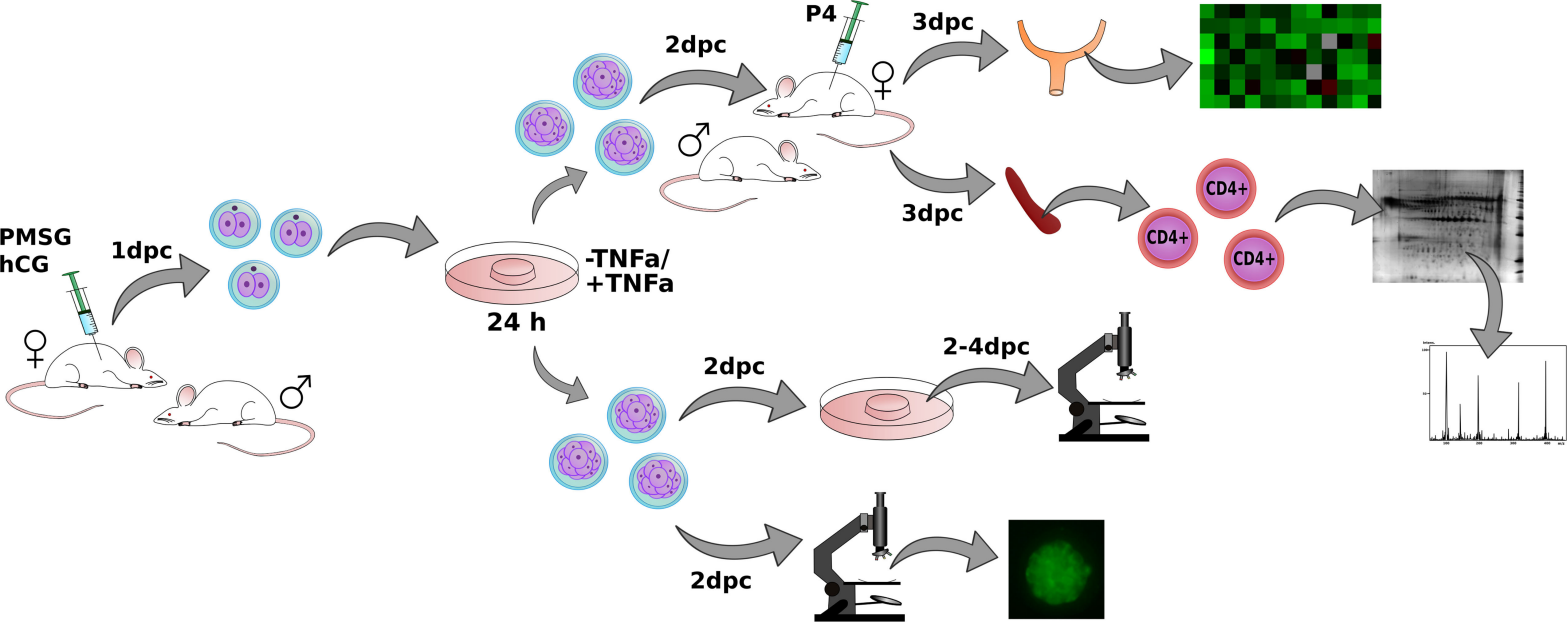
The layout of the entire experiment. Female mouse donors were superovulated by PMSG (Pregnant Mare Serum Gonadotropin) and hCG (human Choriogonadotropin) injections and mated with fertile males. Embryos were collected at the 1st day *post-coitum* (dpc) and cultured for the next 24 h in the presence or without TNFα. Embryos were transferred into progesterone-treated and mated with vasectomized males, female recipients. Uteri and spleens from recipient females were dissected. Gene expression and proteome of CD4^+^ lymphocytes were examined in obtained tissues. A separate group of embryos was examined *in vitro* for checking the developmental progress and phosphatidylserine expression.

Embryo donors were 4–6 weeks old super ovulated female mice. Superovulation was induced by two intraperitoneal injections. First- 5 IU of pregnant mare serum gonadotropin (Folligon, Intervet, Poland) and 46 h later 5 IU of human chorionic gonadotropin (Chorulon, Intervet, Poland). Six hours after the last injection, females were mated with males (of previously confirmed fertility). The day when the vaginal plug was observed was designated as 0 dpc (days *post coitum*). At 1 day of pregnancy, the embryo donor mice were euthanised, and the uteri with oviducts and ovaries were dissected. The oviducts and uteri were checked for the presence of embryos by uterine flushings under microscopic examination on a heating table. Embryos were collected into 400 μL of warm embryo-manipulation medium M2 (Sigma, Poland). From one superovulated female mouse, on average 32 ± 9 embryos were flushed. A layout of the entire experiment is presented in Figure 1.

### Embryo Culture and TNFα Treatment

On the day of collection (1 dpc), morphologically normal embryos (2-cell stage embryos) were pooled from 2 to 3 female donors and randomly distributed into two groups: normal (control) embryos and TNFα-treated embryos. Each drop of the medium contained 8–10 embryos. Collected embryos from the first group were then cultured at 37°C in 30 μL microdrops of the M16 medium (Sigma, Poland) under mineral oil in the atmosphere containing 5% of CO_2_, whereas embryos from the second group were cultured in drops with the addition of 50 ng/ml mouse recombinant TNFα (Sigma Poland). Embryos from both the experimental and control group were used in the experiments on developmental progress, phosphatidylserine expression, and embryo transfer.

### Developmental Progress Assessment of Normal and TNFα-Treated Embryos

Collected embryos (1 dpc) of both groups were cultured as indicated above and inspected under a stereoscopic microscope at 1, and 3 dpc. The numbers of 2-cell embryos (arrested), 3–5-cell embryos, and >5-cell embryos (morula) were determined at each examination. Then morulae were selected and washed 10 times in fresh constitutive changes of M2 medium drops, transferred to a fresh M16 medium, and then observed for two consecutive days, i.e., 3 and 4 dpc. The numbers of morulae, blastocysts, and hatched blastocysts were determined on each day of observations.

### Phosphatidylserine Expression in TNFα- Treated Embryos

Collected embryos (1 dpc) of both groups were cultured up to the morula stage as indicated in section Embryo Culture and TNFα Treatment. Randomly selected morulae from both groups of embryos (*n* = 10) were stained with annexin V and propidium iodide (Annexin-V-FLUOS Staining Kit, Roche Diagnostics, Poland) to confirm the expression of phosphatidylserine as an early sign of apoptosis induction in blastomeres in embryos (28, 30). Briefly, the embryos were transferred to freshly prepared and preheated microdrops of PBS supplemented with Ca^2+^ Mg^2+^, supplemented with bovine serum albumin (BSA; Sigma, Poland). The embryos were washed three times, each time in a fresh microdrop of PBS (100 μL). Afterward, they were stained at room temperature for 10 min with the Annexin-V-FLUOS (Sigma, Poland) solution reagent with the addition of propidium iodide (20 μL of working-strength solution). After that, the embryos were washed in 10 μL microdrops of fresh PBS with Ca^2+^ Mg^2+^. Before the evaluation under a fluorescence microscope (Leica), the embryos were transferred into microdrops of fresh PBS with the addition of an anti-fade solution (Sigma, Poland). The observations were performed immediately after completed staining.

### Female Embryo Recipients and Embryo Transfer

Prior to the transfer of embryos, the oestrous cycle of 6- to 8-week-old recipient females was controlled by Cytocolor staining (MerckMilipore) of vaginal smears. The females in the proestrus phase (before an oestrous phase, in which ovulation takes place) were mated with vasectomised males to induce pseudopregnancy (3). The successful mating was confirmed by the presence of the vaginal plug and was designated as day 0 of pseudopregnancy. At 0 and 1 dpc, the recipient females were treated with a subcutaneous supply of progesterone P4 (Sigma, Poland) at a dose of 0.2 mg per 100 μl per animal to reduce uterine contractions and to prevent the expulsion of transferred material (31).

Embryos from the control and experimental group designated for transfer were cultured for 24 h, i.e., until 2 dpc as indicated in section Embryo Culture and TNFα Treatment. Then, they were carefully washed 10 times in a sequence of fresh warm M2 medium drops. Only morulae with the best morphological score (evenly sized blastomeres with a smooth profile) were selected and subdivided into groups of embryos designated for transfer to female recipient mice. In total 400 embryos (200 in each group) were transferred into recipient females.

At 2 dpc of pseudopregnancy, non-surgical embryo transfer of normal embryos or TNFα-treated embryos was performed using an NSET® Device (ParaTechs, USA). The procedure was performed on females without anesthesia, by inserting the catheter into one of the uterine horns (through the vagina) and with the expulsion of 20 embryos (in 1.8 μl of medium M16) per mouse, according to the manufacturer's instructions. To restrict stressful conditions connected with handling and catheter insertion, overall manipulation time not exceeded 10–20 s. The whole procedure was performed in silence without the presence of bystanders.

At 24 h after the embryo transfer, at 3 dpc, the female recipients were euthanised, and spleens and uteri were dissected. The uteri were analyzed for the presence of embryos (successful transfer confirmation) by uterine flushings under microscopic examination. For further investigation only pregnant (embryo containing) whole uterine horns were preserved. The dissected spleens were preserved for subsequent isolation of CD4^+^ T cells. Dissected uteri were cut and sampled randomly for subsequent RNA isolation and gene expression experiments.

### RNA Isolation and cDNA Synthesis

Collected uterine horns were homogenized and total RNA was isolated as previously described (13). Isolated total RNA was purified using the RNeasy Mini Kit (Qiagen, USA) with an additional step of DNase digestion (DNase I, Qiagen, USA). The resulting RNA (from each group) was combined into three pools consisting of RNA samples from 3, 3, and 4 uteri, respectively. Total RNA (~1 μg from each uterus) was transcribed to cDNA with the use of RT^2^ First Strand Kit (Qiagen, USA). Obtained cDNA was further used for the uterine preimplantation transcriptome investigation.

### Real-Time PCR

Real-time PCR was carried out using the SensiFAST SYBR Hi-ROX Kit (Bioline Ltd., Blirt, Poland) and an ABI StepOnePlus Thermal Cycler System (Applied Biosystems) as previously described (13). In brief, custom RT^2^ Profiler PCR Arrays (SABioscience, USA) used for real-time PCR (*n* = 3 for each group) were designed as previously explained (13). Each array allowed us to identify most relevant genes belonging to 9 signaling pathways: the NFKB pathway, TGFB pathway, P53 pathway, Mitogenic pathway, NOTCH pathway, LDL pathway, WNT pathway, Hedgehog pathway, and JAK–STAT pathway (Table 1). Array used for these experiments, enables analysis of the expression of 84 downstream genes responsive to activation or inhibition of signal transduction pathway. Each array included primers for target genes and a panel of reference genes. The panel consisted of 5 genes: glyceraldehyde 3-phosphate dehydrogenase (*Gapdh*), glucuronidase β (*Gusb*), hypoxanthine guanine phosphoribosyl transferase (*Hprt*), heat shock protein α family class B member 1 (*Hsp90ab1*), and beta actin (*Actb*). *Actb* was automatically designated to normalize PCR Array data according to manufacturer instructions and due to the most stable expression, as its threshold cycle (C_t_) value did not differ by more than a factor of 0.5 between the arrays (data not shown). The transcriptome obtained in the experimental group was compared to that of the control group. Changes in the threshold cycle (ΔC_t_) values were calculated for each gene by subtracting the mean threshold cycle (C_t_) of the *Actb* reference gene from the threshold cycle value of a gene in question. The normalized quantity of transcripts was calculated as 2^−ΔCt^.

**Table 1:** The list of 84 analyzed genes (divided into nine signaling pathways) and their relative expression levels in groups TNE and NE as measured by a real-time PCR array.

**Gene symbol**	**GeneBank**	**Gene description**	**2^**−ΔCt**^ TNE group**	**Standard deviation TNE**	**2^**−ΔCt**^ NE group**	**Standard deviation NE**	**Fold change 2^**−ΔCt**^ TNE/2^**−ΔCt**^ NE**
**NFKB pathway**							
***Birc3***	NM_007464	Baculoviral IAP repeat-containing 3	**0.0053**	**1.72E-05**	***0.0103***	**0.00009**	**0.51**
***Cxcl1***	NM_008176	Chemokine (C-X-C motif) ligand 1	**0.0002**	**1.31E-05**	***0.0011***	**0.00014**	**0.18**
***Icam1***	NM_010493	Intercellular adhesion molecule 1	0.0079	0.000961	0.0088	0.00389	0.9
***Il2***	NM_008366	Interleukin 2	0.0000	2.1E-08	0.0000	0.00001	0
***Myd88***	NM_010851	Myeloid differentiation primary response gene 88	**0.0023**	***0.000201***	**0.0013**	**0.00024**	**1.77**
***Nfkbia***	NM_010907	Nuclear factor of kappa light polypeptide gene enhancer in B cells inhibitor, α	**0.0334**	**0.000166**	**0.0481**	**0.00213**	**0.69**
***Nos2***	NM_010927	Nitric oxide synthase 2, inducible	**0.0027**	**5.69E-05**	***0.0011***	**0.00002**	**2.45**
***Stat1***	NM_009283	Signal transducer and activator of transcription 1, transcript variant 2	**0.0134**	**0.000453**	**0.00353**	**0.0187**	**3.8**
***Vcam1***	NM_011693	Vascular cell adhesion molecule 1	**0.0011**	**3.81E-05**	**0.0021**	**0.00010**	**0.52**
**TGFB pathway**							
***Atf4***	NM_009716	Activating transcription factor 4	0.0309	0.00083	0.0586	0.01619	0.53
***Cdkn1a***	NM_007669	Cyclin-dependent kinase inhibitor 1A (P21), transcript variant 1	0.0013	0.000137	0.0016	0.00012	0.81
***Cdkn1b***	NM_009875	Cyclin-dependent kinase inhibitor 1B	0.0235	0.001351	0.0199	0.00044	1.18
***Cdkn2a***	NM_009877	Cyclin-dependent kinase inhibitor 2A, transcript variant 1	0.0002	2.43E-05	0.0004	0.00015	0.5
***Cdkn2b***	NM_007670	Cyclin-dependent kinase inhibitor 2B (p15, inhibits CDK4)	0.0010	0.00018	0.0010	0.00007	1
***Id1***	NM_010495	Inhibitor of DNA binding 1	0.0226	0.003536	0.0274	0.00110	0.82
***Id2***	NM_010496	Inhibitor of DNA binding 2	0.0440	0.000839	0.0457	0.00370	0.96
***Myc***	NM_010849	Myelocytomatosis oncogene, transcript variant 1	0.0226	0.000277	0.0331	0.01068	0.68
***Sox4***	NM_009238	SRY (sex determining region Y)-box 4	**0.0014**	**7.33E-05**	**0.0009**	**0.00014**	**1.56**
**P53 pathway**							
***Bax***	NM_007527	BCL2-associated X protein	0.0145	0.001641	0.0173	0.00006	0.84
***Cdkn1a***	NM_007669	Cyclin-dependent kinase inhibitor 1A (P21). transcript variant 1	0.0013	0.000137	0.0016	0.00012	0.81
***Egfr***	NM_007912	Epidermal growth factor receptor. transcript variant 2	0.0019	0.000246	0.0246	0.01164	0.08
***Fas***	NM_007987	Fas (TNF receptor superfamily member 6)	0.0057	0.001815	0.0075	0.00453	0.76
***Gadd45a***	NM_007836	Growth arrest and DNA-damage-inducible 45 alpha	0.0097	0.001188	0.0085	0.00021	1.14
***Pcna***	NM_011045	Proliferating cell nuclear antygen	0.0511	0.000501	0.0539	0.00594	0.95
***Rb1***	NM_009029	Retinoblastoma 1	0.0051	0.000255	0.0054	0.00035	0.94
***Tnf***	NM_013693	Tumor necrosis factor	0.0002	8.15E-05	0.0005	0.00031	0.4
***Trp53***	NM_011640	Transformation related protein 53, transcript variant 1	**0.0073**	**6.79E-05**	**0.0089**	**0.00002**	**0.82**
***Mdm2***	NM_010786	Transformed mouse 3T3 cell double minute 2, transcript variant 1	**0.0135**	**0.000672**	**0.0171**	**0.00004**	**0,79**
***Igfbp3***	NM_008343	Insulin-like growth factor binding protein 3	0.1362	0.057531	0.0606	0.00524	2.25
***Atf2***	NM_009715	Activating transcription factor 2, transcript variant 2	0.0199	0.002011	0.0217	0.00132	0.92
***Bax***	NM_007527	BCL2-associated X protein	0.0145	0.001641	0.0173	0.00006	0.84
**MITOGENIC pathway**							
***Egr1***	NM_007913	Early growth response 1	0.0171	0.000259	0.0246	0.01164	0.7
***Egfr***	NM_007912	Epidermal growth factor receptor, transcript variant 2	0.0019	0.000246	0.0017	0.00001	1.12
***Fos***	NM_010234	FBJ osteosarcoma oncogene	0.0057	0.001815	0.0075	0.00453	0.76
***Jun***	NM_010591	Jun proto-oncogene	0.0188	0.000799	0.0231	0.00711	0.81
***Nab2***	NM_008668	Ngfi-A binding protein 2, transcript variant 1	0.0004	0.00019	0.0006	0.00012	0.67
***Map3k1***	NM_011945	Mitogen-activated protein kinase kinase kinase 1	0.0033	2.92E-05	0.0044	0.00062	0.75
***Map3k2***	NM_011946	Mitogen-activated protein kinase kinase kinase 2	0.0100	0.00062	0.0132	0.00179	0.76
***Mapk1***	NM_011949	Mitogen-activated protein kinase 1, transcript variant 1	0.0425	0.003356	0.0436	0.00033	0.97
***Mapk3***	NM_011952	Mitogen-activated protein kinase 3	0.0281	0.001653	0.0271	0.00196	1.04
***Map2k1***	NM_008927	Mitogen-activated protein kinase kinase 1	0.0111	0.001117	0.0099	0.00150	1.12
***Map2k2***	NM_023138	Mitogen-activated protein kinase kinase 2	0.0016	9.83E-05	0.0021	0.00047	0.76
***Atf2***	NM_009715	Activating transcription factor 2, transcript variant 2	0.0199	0.002011	0.0217	0.00132	0.92
***Trp53***	NM_011640	Transformation related protein 53, transcript variant 1	**0.0073**	**6.79E-05**	**0.0089**	**0.00002**	**0.82**
**NOTCH pathway**							
***Ccnd1***	NM_007631	Cyclin D1	0.0201	0.002465	0.0342	0.01177	0.59
***Cd44***	NM_009851	CD44 antigen, transcript variant 1	0.0285	0.00288	0.0413	0.01007	0.69
***Hes1***	NM_008235	Hairy and enhancer of split 1 (Drosophila)	0.0071	0.000701	0.0152	0.00414	0.47
***Hes5***	NM_010419	Hairy and enhancer of split 5 (Drosophila)	0.0004	6.81E-06	0.0003	0.00014	1.33
***Hey1***	NM_010423	Hairy/enhancer-of-split related with YRPW motif 1	0.0018	1.92E-05	0.0016	0.00013	1.13
***Hey2***	NM_013904	Hairy/enhancer-of-split related with YRPW motif 2	0.0008	1.21E-05	0.0008	0.00013	1
***Id1***	NM_010495	Inhibitor of DNA binding 1	0.0226	0.003536	0.0274	0.00110	0.82
***Jag1***	NM_013822	Jagged 1	0.0110	0.001173	0.0142	0.00150	0.77
***Il2ra***	NM_008367	Interleukin 2 receptor, alpha chain	0.0004	4.78E-06	0.0004	0.00008	1
***Notch1***	NM_008714	Notch 1	0.0043	0.000661	0.0039	0.00112	1.1
***Ppard***	NM_011145.3	Peroxisome proliferator activated receptor delta	0.0018	0.000179	0.0017	0.00017	1.06
***Pparg***	NM_011146	Peroxisome proliferator activated receptor gamma, transcript variant 2	**0.0006**	**3.34E-05**	**0.0007**	0.00002	0.86
**LDL patway**							
***Ccl2***	NM_011333	Chemokine (C-C motif) ligand 2	0.0028	0.000353	0.0131	0.00875	0.21
***Csf1***	NM_001113529	Colony stimulating factor 1	0.0030	0.000419	0.0055	0.00278	0.55
***Csf2***	NM_009969	Colony stimulating factor 2 (granulocyte-macrophage)	0.0002	2.41E-05	0.0003	0.00000	0.67
***Sele***	NM_011345	Selectin, endothelial cell	0.0009	2.45E-05	0.0009	0.00009	1
***Selp***	NM_011347	Selectin, platelet	0.0031	5.59E-05	0.0032	0.00056	0.97
***Vcam1***	NM_011693	Vascular cell adhesion molecule 1	**0.0011**	**3.81E-05**	**0.0021**	**0.00010**	**0.52**
**WNT patway**							
***Axin2***	NM_015732	Axin2	0.0066	0.000882	0.0082	0.00153	0.8
***Birc5***	NM_009689	Baculoviral IAP repeat-containing 5, transcript variant1	0.0190	0.000796	0.0136	0.00227	1.4
***Ccnd1***	NM_007631	Cyclin D1	0.0201	0.002465	0.0342	0.01177	0.59
***Ccnd2***	NM_009829	Cyclin D2	0.0133	0.000735	0.0160	0.00200	0.83
***Cdh1***	NM_009864	Cadherin 1	0.0100	0.000315	0.0110	0.00368	0.91
***Jun***	NM_010591	Jun proto-oncogene	0.0188	0.000799	0.0231	0.00711	0.81
***Lef1***	NM_010703	Lymphoid enhancer binding factor 1, transcript variant 1	0.0090	0.000215	0.0075	0.00225	1.2
***Myc***	NM_010849	Myelocytomatosis oncogene, transcript variant 1	0.0226	0.000277	0.0331	0.01068	0.68
***Pparg***	NM_011146	Peroxisome proliferator activated receptor gamma, transcript variant 2	**0.0006**	**3.34E-05**	**0.0007**	**0.00002**	**0.86**
***Tcf7***	NM_009331	Transcription factor 7, T cell specific	0.0065	0.000102	0.0075	0.00083	0.87
***Vegfa***	NM_009505	Vascular endothelial growth factor A, transcript variant 2	**0.0138**	**2.42E-06**	**0.0191**	**0.00146**	**0.72**
***Wisp1***	NM_018865	WNT1 inducible signaling pathway protein 1	**0.0028**	**0.000233**	**0.0039**	**0.00016**	**0.72**
**HEDGEHOG pathway**							
***Bcl2***	NM_009741	B cell leukemia/lymphoma 2, transcript variant 1	0.0095	4.77E-06	0.0100	0.00192	0.95
***Bmp2***	NM_007553	BZne morphogenetic protein 2	0.0008	0.000125	0.0012	0.00032	0.67
***Bmp4***	NM_007554	Bone morphogenetic protein 4	0.0047	6.82E-05	0.0068	0.00176	0.69
***Foxa2***	NM_010446	Forkhead box A2, transcript variant 2	0.0008	3.07E-05	0.0007	0.00027	1.14
***Hhip***	NM_020259	Hedgehog-interacting protein	0.0017	0.000204	0.0016	0.00049	1.06
***Ptch1***	NM_008957	Patched homolog 1	0.0076	0.000214	0.0071	0.00056	1.07
***Wnt2***	NM_023653	Wingless-type MMTV integration site family, member 2	0.0002	4.67E-06	0.0003	0.00001	0.67
***Wnt5a***	NM_009524	Wingless-type MMTV integration site family, member 5A, transcript variant 1	**0.0246**	**0.002775**	**0.0337**	**0.00140**	**0.73**
***Wnt6***	NM_009526	Wingless-type MMTV integration site family, member 6	0.0005	0.000164	0.0006	0.00002	0.83
**JAK/STAT pathway**							
***Cxcl9***	NM_008599	Chemokine (C-X-C motif) ligand 9	**0.0001**	**6.72E-05**	**0.0006**	**0.00025**	**0.17**
***Ccnd1***	NM_007631	Cyclin D1	0.0201	0.002465	0.0342	0.01177	0.59
***Gata3***	NM_008091	GATA binding protein 3	0.0010	1.48E-06	0.0010	0.00012	1
***Il4ra***	NM_001008700	Interleukin 4 receptor, Ralpha	0.0082	0.000112	0.0055	0.00146	1.49
***Irf1***	NM_008390	Interferon regulatory factor 1. transcript variant 1	0.0123	0.000843	0.0149	0.00041	0.83
***Mmp10***	NM_019471	Matrix metallopeptidase 10	0.0123	0.000843	0.0005	0.00021	24.6
***Nos2***	NM_010927	Nitric oxide synthase 2, inducible	**0.0027**	**5.69E-05**	**0.0011**	**0.00002**	**2.45**
***Socs3***	NM_007707	Suppressor of cytokine signaling 3	0.0063	0.000131	0.0046	0.00127	1.37
**Crucial to implantation genes**							
***Hoxa10***	NM_008263	Homeobox A10. transcript variant 1	0.1223	0.00938	0.1143	0.02479	1.07
***Lif***	NM_008501	Leukemia inhibitory factor, transcript variant 1	0.0015	0.000563	0.0008	0.00041	1.88
***Ptgs2***	NM_011198	Prostaglandin-endoperoxide synthase 2	0.0005	9.42E-05	0.0019	9.12E-05	0.25

*Statistically significant differences (Student's t-test, n = 3) in gene expression are boldfaced; p < 0.05*.

### Unsupervised Clustering of RT-PCR Data

Unsupervised hierarchical clustering has been performed using ΔC_t_ as an input. The decision on the optimal number of sample clusters was made based on consensus partitioning implemented in the “cola” Bioconductor package. Partitioning was performed by use of hierarchical clustering. We tested a range of clusters (K) from 2 to 5 across increasing variable subsets (from “10” to “70” in 10 steps) with 20 repeats and random resampling of 80% of samples. Optimal K has been deduced based on inspection of plots generated by the “cola” package including PAC score, Shilouette score, Rand and Jaccard indexes.

### CD4^+^ T-Cell Isolation and Separation

A single-cell suspension of splenocytes was obtained by immediately squeezing the collected spleen through the cell strainer into an ammonium chloride solution (8.3 g/L) enabling erythrocyte lysis. Splenocytes were sorted using the EasySep™ Mouse CD4^+^ T Cell Isolation Kit (StemCell™ Technologies). Flow-cytometric analysis involving staining with a fluorescein isothiocyanate (FITC)-conjugated anti-mouse CD4 antibody (eBioscience) confirmed the purity of the isolated cells. The average purity was 88.0% ± 5.01 of CD4^+^ cells among all the isolated lymphocytes. The average concentration of isolated cells was (3.5 ± 2.45) × 10^6^/ml [(1.0–9.5) × 10^6^ cells/ml per sorting]. Isolated CD4^+^ cells were then separated for membrane lysis and protein separation by 2-dimensional electrophoresis (2-DE).

### 2-DE and Imaging

Sample preparation for 2-DE analysis was performed using the same procedure as previously described (26). Due to low protein content, 7 μg of each sample (two replicates of each individual) was used for the 2-DE separation. The isoelectric focusing (IEF) was carried out on 7 cm IPG strips with nonlinear pH interval 4–7 (Bio-Rad, USA) with the aid of the Protean® IEF Cell (Bio-Rad, USA) using the rapid protocol for the 7 cm strip (3,000 V, 50 μA, 15,000 Vh). Once separated by IEF, the strips were further incubated in the basal equilibration buffer (6 M urea, 0.5 M Tris/HCl pH 6.8, 2% w/v SDS, 30% w/v glycerol) with an addition of 1% (w/v) of dithiothreitol for 15 min. Next, the strips were placed in the same basal buffer with an addition of 2.5% (w/v) of iodoacetamide for another 20 min. After the equilibration process. The proteins covered with negatively charged SDS molecules were separated using SDS-PAGE on the 12% polyacrylamide gels using Protean Plus™ Dodeca Cell™ electrophoretic chamber (Bio-Rad, USA) at 100 V for 120 min at room temperature. Subsequently, gels with resolved proteins were visualized with silver stain according to Chevallet et al. (32). Gel images were digitalized using GS-800™ Calibrated Densitometer (Bio-Rad, USA). Then, protein spots expression patterns on digital images representing both control and experimental conditions were analyzed using PDQuest v. 8.0.1 Advanced (Bio-Rad, USA). The proteins spots that appeared on at least four gels representing the replicate group were further processed. The gel matching was performed on the basis of the master image protein spots distribution pattern. Normalized spots volumes were taken for the quantitative analysis of the protein spots abundance. The experimental and intragroup variability was assessed as a coefficient of variation (CV%). The significance level (*P*-values below or equal to 0.05 were assumed to indicate significance) was used to select differentially expressed protein spots. The differences between the control and experimental groups were expressed as arithmetic mean and on that basis, fold change ratio was calculated. The molecular weights of protein spots were calculated against the Precision Plus Protein™ Standard Plugs (Bio-Rad, USA) mass ruler. Experimental *pI*s were calculated on the basis of pH gradient distribution of the IPG strips. The qualitative gels served as a source of protein spots harvested for protein identification. For this purpose, pooled protein samples (~70 μg of lymphocyte total protein) were separated in 2 replicates. Protein spots detection was performed using colloidal Coomassie stain according to the methodology described by Pink et al. (33).

### Mass Spectrometry

Significantly altered spots were manually excised from the preparative 2-D gels, destained and trypsin digested according to the protocol previously described by Chełmońska-Soyta et al. (15). The obtained peptide mixtures were then extracted from the gel pieces with 100% acetonitrile (ACN). Next, the equal volumes (1:1 μl) of peptide mixtures and CHCA matrix solution (5 mg/ml α-cyano-4-hydroxy-cinnamic acid, 0.1% v/v trifluoroacetic acid in 50% v/v ACN) were deposited on the targets of MALDI-MSP AnchorChip™ 600/96 plate (Bruker Daltonics, Germany). External calibration within the peptides mass range between 700 and 3,200 Da was performed using peptide mass standard II (Bruker Daltonics, Germany). Mass spectra acquisition was carried out in the positive ionization with reflection mode using Microflex™ MALDI TOF mass spectrometer (Bruker Daltonics, Germany). Peptide mass lists were compared with *in silico* protein digestion data deposited in the protein sequence databases (NCBI and Uniprot) using MASCOT search engine. The following query criteria were applied: cysteine modification, methionine oxidation, 150 ppm peptide mass tolerance, one trypsin missed cleavage site. Probabilistic scoring algorithm was used to assess the significance of protein identification followed by the minimal 20% of sequence coverage value. Determination of subcellular protein distribution was analyzed using Euk-mPLoc 2.0 (http://www.csbio.sjtu.edu.cn/bioinf/euk-multi-2/).

### Statistical Analysis

Statistical analysis was carried out in the R program. Shapiro test was used to assess the shape of the data distribution. The Mann–Whitney test (non-parametric), Student's *t*-test, or χ^2^ test (embryo developmental assessment) was performed based on the shape of the data distribution. Data with *P*-values below 0.05 were considered significant.

## Results

### Embryo Development Assessment

The culture of collected embryos up to the morula stage in the presence of TNFα revealed no significant differences (χ^2^ test, *p* > 0.05) in the development rate in comparison to untreated embryos (Table 2). To check the developmental competency of *in vitro*–obtained morulae, the embryos were examined at consecutive time points until the stage of the hatched blastocyst (4 dpc). The developmental rates of washed morulae were similar between the two groups of embryos (Table 3).

**Table 2:** The developmental rate of embryos collected from mice at 1 dpc and cultured *in vitro* for 24 h in the presence or absence of TNFα (χ^2^ test *p* ≥ 0.05).

	**2-cell embryos (arrested)**	**3-5-cell embryos**	**>5-cell embryos (morula)**
Normal embryos *n* = 60	7 (11.66%)	38 (63.33%)	15 (25%)
TNFα-treated embryos *n* = 60	11 (18.33%)	39 (65%)	10 (16.66%)

**Table 3:** The developmental rate of morulae obtained at 2 dpc after completed cultivation of embryos in the presence or absence of TNFα. Compacted morulae were selected, washed 10 times, and transferred to a fresh culture medium and then examined on three consecutive days (χ^2^ test *p* ≥ 0.05).

	**2 dpc morula**	**3 dpc blastocyst**	**4 dpc hatched blastocyst**
Normal embryos *n* = 30	30 (100%)	29 (96.66%)	19 (63.33%)
TNFα-treated embryos *n* = 30	30 (100%)	28 (93.33%)	18 (60.00%)

### Phosphatidylserine Expression

**Figure 2:**
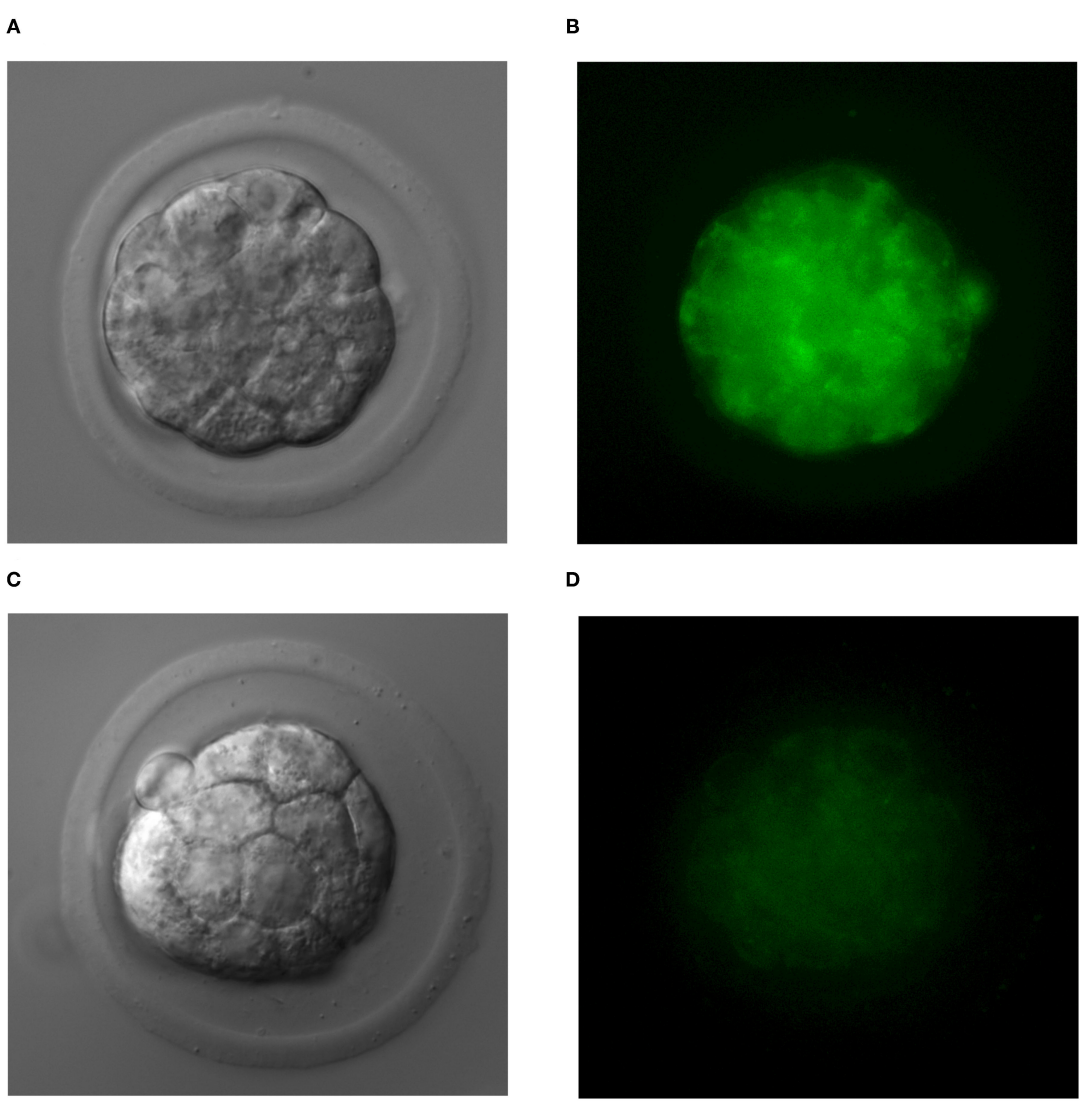
The uniform pattern of phosphatidylserine expression in blastomeres of TNFα-treated embryos **(A,B)** and normal embryos **(C,D)**.

As in our previous experiments (30), we confirmed the expression of phosphatidylserine using FITC-labeled Annexin V in all the examined TNFα-treated embryos, whereas it was not detectable in embryos of the control group. We did not observe PI staining in nuclei of examined embryos. In several TNFα-treated embryos, expression of phosphatidylserine was observed in single blastomeres; however, in most of the examined embryos, a uniform pattern of staining was recorded (Figure 2).

### Real-Time PCR

Real-time PCR analysis resulted in significantly altered (*p* < 0.05) expression of 16 genes. The expression of 3 of these genes was up-regulated and the remaining 13 were down-regulated in the TNE group compared to the NE group. Table 1 presents differentially expressed genes assigned to the nine selected pathways. All the statistically significant down-regulated genes belonged to eight out of nine investigated pathways [NFKB, WNT, Janus kinase (JAK)/Signal Transducer and Activator of Transcription (STAT), protein 53 (P53), HEDGEHOG, NOTCH, low-density lipoprotein (LDL), and MITOGENIC] with 43.8 and 18.7% belonging to NFKB and WNT pathways, respectively. Taking into consideration the number of significantly altered, i.e., down- and up-regulated genes in each pathway as compared to all the genes in the pathway, the most affected pathway was NFKB, with 87.5% of differentially expressed genes (Table 1). The percentage of differentially expressed genes in the remaining pathways ranged from 7.7% (Mitogenic pathway) to 25% (WNT pathway). In addition to the nine analyzed signaling pathways, three genes relevant to the preimplantation process were also analyzed: homeobox A10 (*Hoxa10*), leukemia inhibitory factor (*Lif* ), and prostaglandin-endoperoxide synthase 2 (*Ptgs2*). The transfer of embryos cultured in the presence of TNFα caused a statistically significant decrease in *Ptgs2* (*p* < 0.05) gene expression in the TNE group in comparison with the NE group (Table 1).

### Clustering Analysis

**Figure 3:**
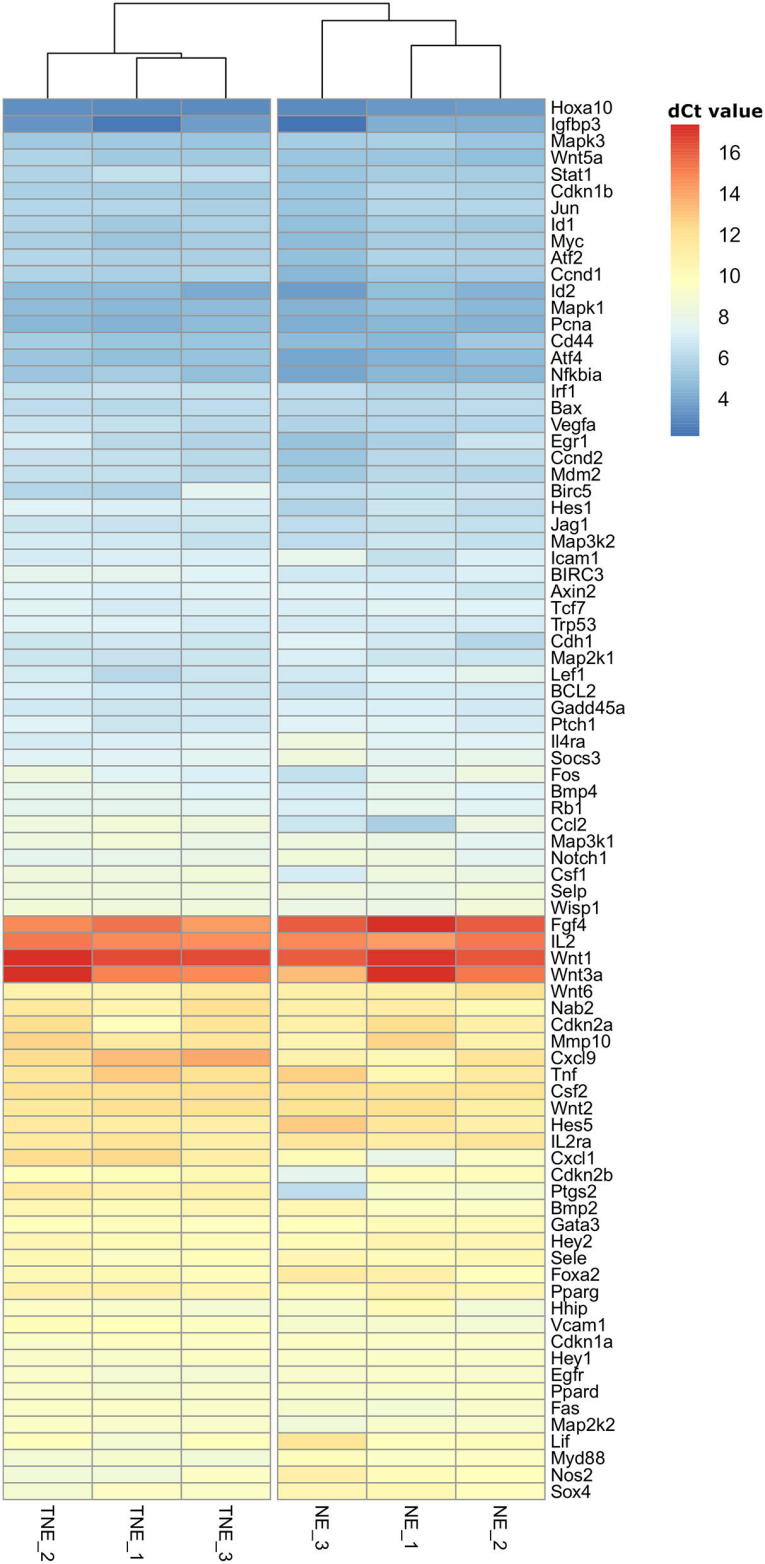
Hierarchical clustering heatmap of mRNA transcripts of nine signal transduction pathways differentially expressed in recipients' uteri of normal (NE) and TNFα- (TNE) treated embryos. Gene expression level is color coded.

Unsupervised clustering reveals clear separation of NE and TNE groups, which confirms that TNFα-treated embryos influence the expression of selected genes (genes are grouped in 3 groups: highly expressed, intermediate expression, and low expression). Results of clustering and separation of NE and TNE groups are presented on the heatmap (Figure 3).

Consensus partitioning revealed *K* = 2 as the most stable number of clusters with clear inclusion of NE and TNE groups into separate clusters. Consequently, the separation of NE and TNE groups confirmed that TNFα influences the expression of selected genes.

### Differentially Expressed Proteins of Splenic CD4^+^ T Lymphocytes in Pregnant Females After the Transfer of Normal and TNFα-Treated Embryos

**Figure 4:**
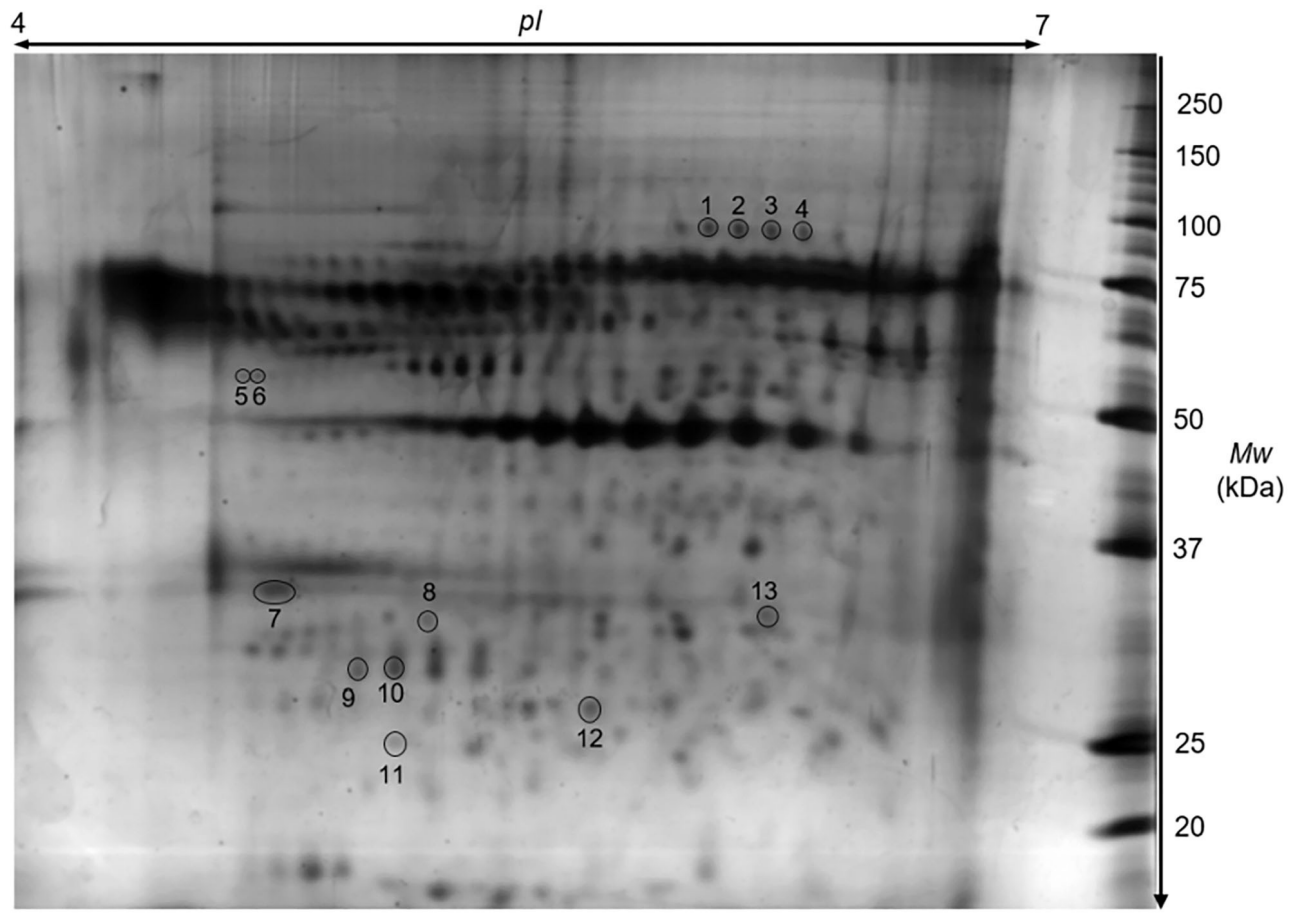
A representative proteomic 2D map of splenic CD4^+^ T cells. The 2-DE gel illustrates a Coomassie-stained protein pattern. Spot numbers correspond to the numbers in Table 4.

Proteins isolated from CD4^+^ T lymphocytes-and obtained from female mice after the transfer of TNFα-treated and normal embryos-were subjected to 2-DE with subsequent mass spectrometric identification of spots showing statistically significant changes. A representative 2-DE map of the experimental groups is presented in Figure 4. As a result of the detection images of 2-DE gels, we revealed the presence of 135–157 protein spots. The coefficient of variation (CV) of the replication ratio in different groups was as follows: for group TNE, 66.28%; for group NE, 67.44%. Thirteen protein spots in lymphocytes of recipients of TNFα-treated embryos were found (using bioinformatics software) significantly altered in comparison with the control group (Table 4).

**Table 4:** The list of differentially expressed proteins of splenic CD4^+^ T cells between mice after the transfer of TNFα-treated embryos in comparison to mice after the transfer of biologically competent embryos (Student's *t-*test *p* ≤ 0.05).

**No**	**Protein name**	**Protein abbreviation**	**Fold change**	**Accession number**	**Peptides matched**	**Sequence coverage (%)/mascot score**	**Theoretical pI/Mr (pH/kDa)**	**Calculated pI/Mr (pH/kDa)**	**Subcellular localization**
1	Heat shock cognate 71kDa protein	HSP70	2.84	Q3U9G0	14	32/109	5.37/71.05	6.1/97.8	Nucleus
2			3.10		11	26/80	5.37/71.05	6.2/97.8	Nucleus
3			3.35		29	49/203	5.37/71.05	6.3/96.2	Nucleus
4			2.63		10	24/73	5.37/71.05	6.4/95.4	Nucleus
5	Lymphocyte – specific protein 1	LSP1	0.51	Q8CD28	8	36/68	4.77/36.81	4.7/56.6	Nucleus
6			0.59		8	35/70	4.77/36.81	4.7/56.6	Nucleus
7	Tropomyosin alpha-3 chain	TPM3	1.39	P21107	11	33/99	4.75/29.02	4.8/34.1	Cytoskeleton
8	Interleukin – 24	IL24	1.92	Q925S4	5	27/61	9.22/20.97	5.2/31.9	Extracellular
9	Rho GDP – dissociation inhibitor 2	GDIR2	2.26	Q61599	7	54/70	4.97/22.89	5.0/28.8	Cytoplasm
10			1.88		6	83/54	4.97/22.89	5.1/29.0	Cytoplasm
11	Peroxiredoxin – 2	PRDX2	1.39	Q61171	5	42/61	5.2/21.94	5.1/25.0	Cytoplasm/ Mitochondrion
12	ATP synthase subunit d, mitochondrial	ATP5H	1.24	Q9DCX2	6	50/59	5.52/18.79	5.9/28.2	Mitochondrion
13	Semaphorin – 7A	SEMA7A	2.42	Q9QUR8	12	22/59	7.83/75.97	6.2/32.2	Extracellular

**Figure 5:**
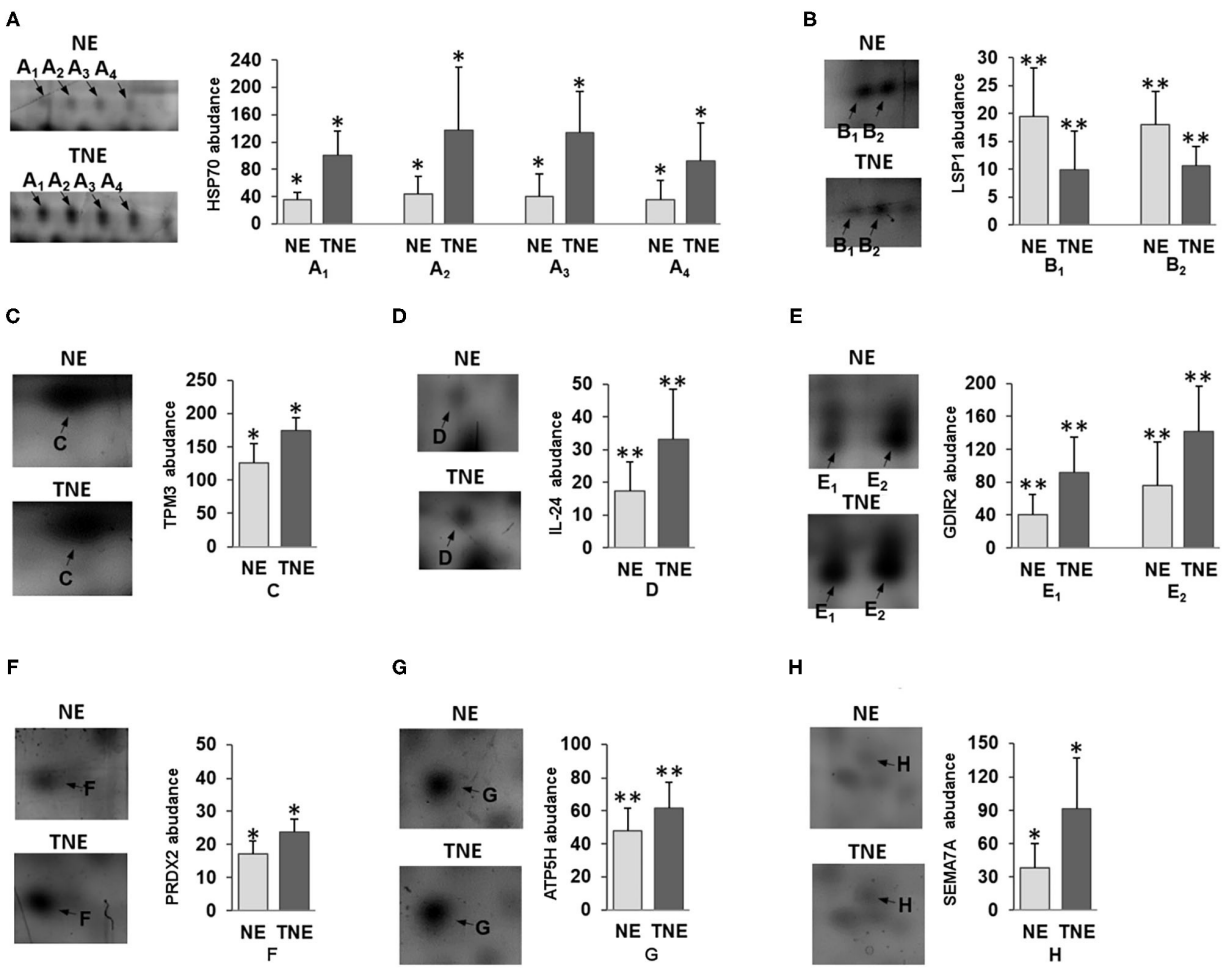
The graphic trend for spots with a variable expression of **(A)** HSP70, **(B)** LSP1, **(C)** TPM3, **(D)** IL-24, **(E)** GDIR2, **(F)** PRDX2, **(G)** ATP5H and **(H)** SEMA7A proteins between TNE (dark gray bars) and NE group of mice (light gray bars). The data concerning the protein expression are given as arithmetic mean with SD (group *n* = 6). Student's *t*-test *p < 0.05, **p < 0.01.

Detailed properties and functions of the identified proteins according to the Swiss-Prot database are listed in Table 4. The adjustment for all the identified proteins was higher in splenocytes isolated from animals after the transfer of the embryos exposed to TNFα. Out of the 8 proteins (represented by 13 protein spots including isoforms of 3 proteins) of differential expression, 7 showed up-regulation [HSP70 (2.84-fold), TPM3 (1.39-fold), IL-24 (1.92-fold), GDIR2 (2.26-fold), PRDX2 (1.39-fold), ATP5H (1.24-fold), SEMA7A (2.42-fold)], and one was down-regulated (LSP1: 0.51-fold). Protein spots with differential expression are marked on the protein map (Figure 4). A graphic trend for spots with differential expression is shown in Figure 5.

## Discussion

The embryo-maternal dialogue during preimplantation pregnancy still involves many unresolved issues. The processes crucial for the maintenance of pregnancy such as genetic abnormal embryo selection, early immune recognition (34), induction of split tolerance (35), and epigenetic modification of embryo gene expression (36), are still not well-understood or are debated. However, it is known that the maternal organism needs to make a quick decision about the fate of pregnancy, because maintaining a pregnancy with a low development potential may result in a miscarriage or low birth weight (37). We showed that TNFα-treated embryos transferred into the uterus of the recipient female mice induce differential signals in mother tissues in comparison with untreated embryos. According to Fabian et al. (28) and Kawamura et al. (38), mouse embryos can produce TNFα and they express the receptors for this cytokine. However, other papers have shown that embryos are sensitive to the increased concentration of TNFα in their vicinity (28, 29). Embryos responded to higher levels of this cytokine by increased blastomeres' apoptosis and decreased proportion of implanted blastocyst. However, based on microscopical observation, such TNFα treatment did not compromise *in vitro* development of mice embryos until the blastocyst stage. On the other hand, an increased concentration of TNFα changed their metabolism by inducing a transient process of apoptosis. Thus, both metabolic changes and impaired implantation signals may influence the process of maternal recognition of TNFα-treated embryos despite the fact of natural production of TNFα by embryos *per se*. In this paper, we confirmed apoptosis induction and unchanged *in vitro* developmental potential of TNFα- treated embryos. Here, we utilize two different methodological approaches for the detection of early embryonic signals. The descriptive analysis showed us that both in local and peripheral compartment embryos of differential biological quality are differentially recognized.

To gain insight into the differences between the expression of genes regulated by all signal transduction pathways, data from all expressed genes in the uteri of NE and TNE groups were used in the cluster analysis. The obtained heatmap clearly suggests differential expression of examined genes between samples of two studied groups. Thus, at the preimplantation stage of pregnancy, a mother can distinguish the differential biological potential of developing embryos at the local level. Moreover, it is worth noting that the majority of examined genes were of low or moderate expression level. This confirms our previous observation on negative regulation of the expression of genes regulated by pathways of signal transduction in the murine uterus in preimplantation pregnancy (13). Similar observations were performed in bovine species where at the 6th and 13th day of normal pregnancy the majority of uterine genes with differential expression compared to non-pregnant females were down-regulated (20). On the other hand, in studies on the endometrial transcriptional activity at the 18th and 20th day of pregnancy, transferred cloned bovine embryos, which are characterized by limited biological potential, differently stimulated the transcriptional activity of genes in the uterus in compared to normal pregnancy. The majority of influenced genes are mainly involved in the immune response and metabolism (17, 39). Direct comparison with studies focusing on endometrial response cannot be considered, as here we analyzed transcriptome from the whole uterus, which could be some limitation of our study. Future studies utilizing separated uterine layers would be beneficial to explore in which tissues gene expression alteration was mostly driven by the presence of TNFα-treated embryos.

Here, we have shown that among the nine analyzed signal transduction pathways, the NFKB pathway (with 87.5% of differentially expressed genes) was the most consistently down-regulated. The involvement of the NFKB pathway in the activity of uterine cells in the peri-implantation period of pregnancy is not well-explored. Nakamura et al. showed that in the pregnant uterus, the NF-kB DNA-binding activity is detectable at 1.5 dpc, and further strengthened and reached a peak at 6.5 dpc, when the implantation of the conceptus is completed (40). The NFKB pathway is regulated in various cells (epithelial, stromal, and immune cells) both by exogenous and endogenous ligands. This pathway could be activated in different manners: canonical, non-canonical, and atypical (41–43). Among the genes regulated by the canonical pathway, statistically, significant differences in expression of *Nfkbia* (Nucleus polypeptide gene enhancer in B cells inhibitor, α), *Myd88* (myeloid differentiation primary response gene 88), *Stat1* (transcript variant 2), and *Nos2* (nitric oxide synthase 2, inducible) were observed. Among the genes regulated by the non-canonical pathway: *Birc3* (baculoviral IAP repeat-containing 3), *Cxcl1* [chemokine (C-X-C motif) ligand 1], *Vcam1*, and *Nos2* showed differential expression between the examined groups. The increased expression of the *Myd88* gene in mice is evenly distributed in the uterine luminal epithelium on day 4 of pregnancy, but its expression was not detected at 2 and 3 dpc (16). This result is consistent with our observation in a previous experiment in which uterine expression of *Myd88*, in natural pregnancy, at 3.5 dpc before implantation did not differ from that in pseudopregnant mice (13). In the present study, significantly increased expression of genes *Myd88* and *Nos2* and decreased expression of the *Nfkbia* pathway inhibitor suggests activation of the canonical pathway.

On the other hand, expression of genes mainly regulated by the non-canonical pathway was down-regulated in the uteri of mice after transplantation of TNFα-treated embryos. Decreased expression of genes of chemokine ligand *Cxcl1* and the *Vcam1* adhesion molecule suggests that the traffic of immune cells within the preimplantation pregnant uterus may be differentially regulated in recipients of normal and TNFα-treated embryos. Additionally, the NFKB pathway has control over the pregnancy implantation window, which is manifested by the regulation of recognition and maintenance factors like *Lif*, *Ptgs2*, and *Hoxa10*, which are essential for pregnancy (44). In our experiments, activation of the NFKB pathway did not correlate with the expression of genes *Lif* and *Hoxa-10*, although there was a correlation with diminished expression of *Ptgs2*. Prostaglandin 2 synthase (PTGS2, encoded by *Ptgs2*) expression is detectable during pregnancy in mice in the luminal epithelium and stroma at the time of blastocyst implantation (45), and even in the preimplantation period, i.e., 3.5 dpc (3). The blastocyst presence is a determinant of the induction of *Ptgs2* during pregnancy before implantation (46, 47). Thus, TNFα treated embryos may provoke differential regulation of gene expression of this importance, for the process of implantation, molecule. We believe that results of our study showing differential gene expression presumably influenced by the embryo biological potential, will bring new insight to the field of early embryo-maternal dialogue. Notwithstanding, due to the limitations resulting from the small sample number (*n* = 3 pooled uterine material), the results presented in Table 1 should be considered as indicative and further in depth studies should be performed.

Similar to the aforementioned local response, the maternal peripheral immune system is sensitive to the presence of a developing embryo. Several previous results obtained in mice, humans and ruminants indicated that peripheral leukocytes changed their phenotype during preimplantation pregnancy. During *in vitro* experiments with human pregnancy, β-human chorionic gonadotropin exerts an immunomodulatory effect on peripheral blood lymphocytes by decreasing production of IFN-gamma and IL-2 and by increasing production of IL-10 (48, 49). On the other hand, the last experiments performed by Pflitsch et al. showed that blood monocytes from pregnant women can be distinguished from monocytes from non-pregnant women based on their phenotype. Moreover, monocyte surface molecules' expression was correlated with serum β-hCG level (50). In ruminants the effect of an embryo-derived early pregnancy signal - IFN-tau is also observed at the periphery. In heifers, on the 18th day of pregnancy increased expression of interferon-stimulated genes in peripheral leukocytes is related to pregnancy outcome (51, 52).

Here, we demonstrated that in mice, the proteome of splenic CD4^+^ T lymphocytes is different in female recipients of normal embryos in comparison with recipients of TNFα-treated embryos. Proteins with altered expression were classified as (1) proteins influenced by cell stress, (2) those involved in the regulation of cytoskeleton stabilization and lymphocyte motility, and (3) proteins of an immunomodulatory function. Two cell stress–sensing proteins, i.e., heat-shock protein (HSP) and its isoforms and peroxiredoxin 2 (PRDX2), were significantly up-regulated after the transfer of TNFα-treated embryos. It is known that an increased amount of heat shock protein 70 (HSP70) in the lymphocytes (53) and high concentrations of anti-HSP70 antibodies in the serum of pregnant women are considered prognostic factors of a birth outcome, in particular, delivery of new-borns with birth defects (54). Therefore, increased expression of HSP70 in peripheral lymphocytes of females bearing TNFα-treated embryos confirmed the indicator role of this protein for a risky pregnancy. On the other hand, the observed enhanced expression of peroxiredoxin II (*p* < 0.05) in splenic T lymphocytes of recipients transferred with TNFα-treated embryos is suggestive for the importance of protection from oxidative stress (55). Moreover, injection of an anti-PRDX2 neutralizing antibody increases NK-cell cytotoxicity and raises the fetal absorption rate in an abortion-prone mouse model (56). Thus, alteration of the levels of HSP70 and PRDX2 in T lymphocytes of the TNE group indicates that cells beyond the reproductive tract are sensitive to stress-inducing signaling from impaired embryos at the preimplantation stage of pregnancy.

Transplantation of TNFα-treated embryos also up-regulated the proteins with immunoregulatory properties in the lymphocytes of female recipients, i.e., interleukin 24 (IL-24) and semaphorin 7a (SEMA7A). To our knowledge, we are the first to report IL-24 presence in peripheral T lymphocytes at such early stages of pregnancy. In murine models, IL-24 has been shown to down-regulate Th1 responses and to up-regulate Th2 responses, indicating its possible positive role in early pregnancy outcomes (57). However, the exact role of IL-24 in pregnancy needs to be proven. In the TNE group, the increased level of SEMA7A, which plays a critical role in the negative regulation of T-cell activation and function, may also point to the engagement of CD4^+^ T cells in the response to embryo signals. It is not clear how the altered expression of the two above-mentioned proteins participates in the modulation of an immune response during preimplantation pregnancy after transplantation of impaired embryos. Nevertheless, it may be assumed that the peripheral immune system can differentially respond to the presence of low-quality embryos. Another group of proteins that showed expression changes in mice after the transfer of TNFα-treated embryos comprised: the α tropomyosin 3 chain (TPM3), dissociation inhibitor Rho-GDP 2 (GDIR2), ATP synthase d subunit (ATP5H), and protein specific for lymphocytes 1 (LSP1). As in our previous studies (26), we confirmed their differential expression in response to the presence of embryos of different quality. Therefore, the migration activity of splenic CD4^+^ T lymphocytes may depend on the quality of the embryonic signal, especially during the development of the embryo under conditions of stress and the risk of pregnancy loss.

Taking into consideration all the aforementioned results, further studies should be performed to elucidate the influence of differential activation of proinflammatory signal transduction pathways locally in the uterus on the immunophenotype of peripheral lymphocytes and, most importantly, how it may influence on a maternal organism in deciding about the fate of pregnancy.

## Conclusion

Our results suggest that the maternal organism can distinguish embryos with diverse developmental potentials already in the preimplantation period both in the local and peripheral compartment. Our study provides new insight into an embryo–maternal communication at the early stages of pregnancy. The nature of the signals capable of modulating cellular changes in distant compartments remains elusive, although it is tempting to speculate that these signals are generated differentially in response to normal and developmentally impaired embryos (58). We also suggest that peripheral lymphocytes in particular can serve as biological sensors of embryo quality upon implantation. Nevertheless, further detailed studies on the expression of activated proteins in signal transduction pathways, the significance of protein expression in CD4^+^ lymphocytes activity, and their mutual interactions during normal and compromised preimplantation pregnancy are needed.

## Data Availability Statement

The datasets presented in this study can be found in online repositories. The names of the repository/repositories and accession number(s) can be found here: Repository of the Wrocław University of Environmental and Life Sciences, Poland https://arche.upwr.edu.pl/index.php/s/OxfEzcZaMokEzdZ/download.

## Ethics Statement

The animal study was reviewed and approved by Local Ethics Committee for Experiments on Animals at the Hirszfeld Institute of Immunology and Experimental Therapy in Wroclaw.

## Author Contributions

KB-M: conceptualization, methodology, investigation, and writing original draft. AK: methodology, visualization, investigation, and writing original draft. AL, AH, and MO: investigation and editing original draft. PK: visualization and writing-review and editing. AG, AS, and PD: methodology and writing-review and editing. DL: visualization and writing original draft. AC-S: conceptualization, resources, funding acquisition, and writing original draft. All authors have read and agreed to the published version of the manuscript.

## Conflict of Interest

The authors declare that the research was conducted in the absence of any commercial or financial relationships that could be construed as a potential conflict of interest.
